# Changes in weight distribution and trends in obesity among children and adolescents in East Asia: Insights from NCD-RisC data

**DOI:** 10.1371/journal.pone.0310646

**Published:** 2024-11-12

**Authors:** Yong Hee Hong, Sujin Park, Minsoo Shin, Sochung Chung, Jahye Jung, Ah-Ram Sul, Yoon Lee

**Affiliations:** 1 Department of Pediatrics, Soonchunhyang University Bucheon Hospital, Soonchunhyang University College of Medicine, Bucheon, Korea; 2 Department of Health Sciences, Graduate School, Korea University, Seoul, Korea; 3 Department of Pediatrics, Korea University Ansan Hospital, Ansan, Korea; 4 Department of Pediatrics, Konkuk University School of Medicine, Seoul, Korea; 5 Department of Pediatrics, Konkuk University Medical Center, Seoul, Korea; 6 Division of Healthcare Research, National Evidence-based Healthcare Collaborating Agency, Seoul, Korea; 7 Department of Pediatrics, Korea University Medical Center Anam Hospital, Seoul, Korea; Indian Institute of Public Health Gandhinagar, INDIA

## Abstract

Pediatric obesity is a serious global health challenge. In East Asia, rapid socioeconomic changes have increased obesity rates. This study examines weight distribution and obesity trends in East Asian children using WHO criteria. Data from the Non-Communicable Disease Risk Factor Collaboration was used to analyze weight categories (thinness, normal weight, overweight, and obesity) among children aged 5 to 19 in China, Japan, South Korea, and Taiwan. Data were collected through probabilistic sampling and national surveys and classified using WHO BMI standards. Age standardized prevalence rate enabled cross-country comparisons for boys and girls from 2010 to 2022. Statistical methods included arithmetic statistics, linear regression, and time series analysis using the ARIMA model, with SAS 9.4 and SPSS for analysis. Significant trends were found (p for trend < 0.05). Taiwan and South Korea showed increased thinness, while China and Japan showed decreases. Normal weight prevalence declined, notably among South Korean boys. Overweight and obesity rates rose, especially among South Korean boys and Chinese girls. Japan’s rates remained stable, while Taiwan showed minor fluctuations. Boys had higher overweight and obesity rates than girls in all countries. The highest rates were among children aged 10 to 11 years. East Asia, particularly South Korea and China, has seen rising obesity rates. Increasing thinness in South Korea and Taiwan also requires attention. The decline in healthy-weight children is concerning. Interventions should target children before ages 10 to 11. Urgent, tailored public health interventions are needed.

## Introduction

Childhood and adolescent obesity is one of the most serious global public health challenges affecting every country in the world [1]. There has been increasing awareness on the health of children and adolescents because growth and development during childhood and adolescence significantly impact lifelong health and well-being. In particular, obesity during childhood and adolescence has been shown to increase the risk of overweight, obesity, and various non-communicable diseases in adulthood, and it might also contribute to children’s poor educational outcomes [2].

As global obesity rates continue to rise, certain regions have experienced particularly rapid changes, underscoring the need for focused public health interventions. The regions with the largest increases in such incidences are East Asia, the Middle East and North Africa, South Asia, and other high-income English-speaking areas [3]. The rapid economic development and urbanization in East Asian countries like China (People’s Republic of China, PRC), Japan, South Korea, and Taiwan (The Republic of China, ROC) have led to profound shifts in dietary habits (e.g., increasing consumption of fast food, processed foods, sugary beverages) and physical activity levels (e.g., increased screen time, reduced physical activity due to urban environments) [4]. These changes have contributed to growing prevalence rates of overweight and obesity among children in this region. Simultaneously, other weight-related issues, such as thinness, continue to persist, adding complexity to the nutritional and health challenges faced by some countries’ children [5]. While these East Asian countries undergo similar societal transformations, disparities exist in cultural awareness and policy implementations within each nation. However, cross-country comparative research has been limited.

To better understand the current trends and challenges, it is critical to examine the weight distribution among children across East Asia, including the prevalence rates of thinness, normal weight, overweight, and obesity. Due to differences in obesity definitions and country-specific growth charts, direct comparisons of obesity prevalence across countries may not be appropriate. This study sought to analyze the weight trends among children aged 5 to 19 in China, Japan, South Korea, and Taiwan using data from the Non-Communicable Disease Risk Factor Collaboration (NCD-RisC). By utilizing World Health Organization (WHO) criteria and applying statistical methods, this research will provide insights into the evolving patterns of pediatric weight status in the region, highlighting areas of concern and the need for targeted public health interventions. This study also aims to inform policymakers, healthcare providers, and educators involved in government policy on food marketing and physical activity, community-level initiatives, and school-based programs about the importance of addressing the trends of pediatric obesity and thinness, emphasizing the need for comprehensive, region-specific strategies to improve the health and well-being of East Asian children.

## Materials and methods

### Data and subjects

We used the data of the Non-Communicable Disease Risk Factor Collaboration (NCD-RisC), a network of health scientists around the world that provides rigorous and timely data on major risk factors for non-communicable diseases for all of the world’s countries (NCD Risk Factor [6]). Data were obtained from national measurement surveys that were publicly available. With the help of the WHO regional and country offices, population-based survey data were identified and accessed from national health and statistical agencies. These data were collected using a probabilistic sampling method with a defined sampling frame and included population samples at the national, subnational, or community level [5]. Estimates of the prevalence rates for thinness, normal weight, overweight, and obesity, broken down by region, country, and sex, were sourced from publicly available databases at www.ncdrisc.org.

This study compares the prevalence rates of weight groups among children and adolescents aged 5 to 19 in East Asia, focusing on China, Japan, South Korea, and Taiwan.

The Institutional Review Board of the National Evidence-based Healthcare Collaborating Agency (NECA) in Korea approved the current study (IRB No. NECAIRB23-015 approval date 25-July-2023). The data used in this study was sourced from public databases, and informed consent was waived from the IRB (date of data assessment: 6-March-2024). The authors were unable to access any information that could identify individual participants during or after data collection.

### The definition and measurement

The weight groups were divided into four categories based on standard deviations (SD) of body mass index (BMI): thinness, normal weight, overweight, and obesity. The respective weight groups were defined as follows: more than 2 SDs below the median of the WHO growth reference, from 2 SDs below the median to 1 SD above the median, 1 SD to 2 SDs above the median, and more than 2 SD above the median.

The prevalence rate of weight groups was estimated based on the proportion of each group within the total population. To enable comparisons among countries, Age Standardized Prevalence Rates (ASPR) from ages 5 to 19 were utilized, excluding age-specific prevalence data. The study data were analyzed separately for girls and boys to account for biological differences, and a span of 12 years (2010 to 2022) was examined to observe trends in prevalence.

### Statistical analysis

Summary data were compiled to assess the current prevalence based on weight groups in each country. Arithmetic statistics were used to confirm the absolute difference in prevalence between 2010 and 2022 and assess the degree of changes in weight groups over the years.

Linear regression analysis was conducted to measure beta coefficients for determining the amount of change, and a time series analysis using the ARIMA (autoregressive integrated moving average) model was used to test the significance of the trend, as indicated by the p for trend. A p-value threshold of 0.05 was set for testing the significance of the trend, and the significance was determined based on the 95% confidence interval. All statistical calculations were conducted with SAS 9.4 software, and the corresponding visual outputs were produced using SPSS 25.

## Results

### Prevalence trend in East Asia

**Fig 1:**
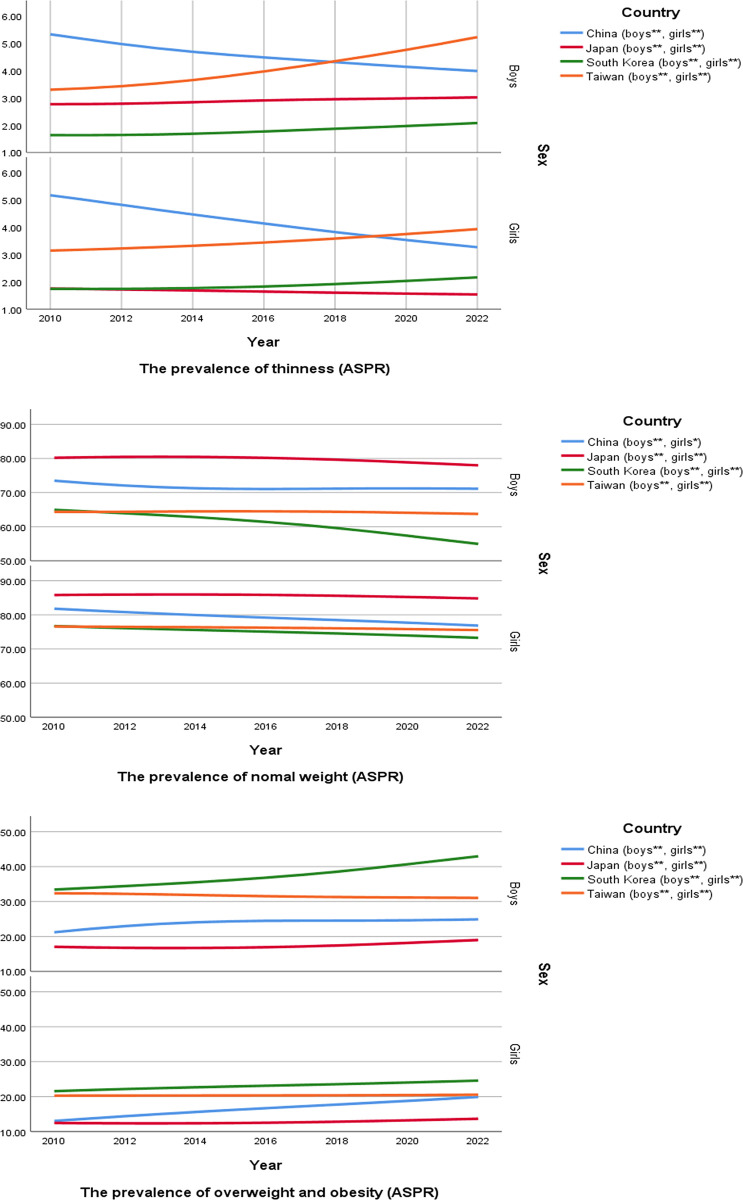
Trends in prevalence rates of thinness, normal weight, overweight including obesity among East Asian children and adolescents between 2010 and 2022.

We analyzed the trends in the prevalence across all countries by gender and weight group over the past 12 years. These trends are found to be statistically significant (p for trend < 0.05) (Fig 1).

The trend shows an increase in thinness in Taiwan and South Korea and a decrease in China and Japan. Particularly, the increase in thinness in Taiwan was noticeably pronounced. The proportion of normal weight has been decreasing in all countries, with a sharp decline observed among South Korean boys since 2018. The decrease in normal weight suggests a potential increase in thinness or overweight and obesity. Overweight and obesity have been on the rise in general, with a particularly rapid increase among South Korean boys and Chinese girls. In contrast, Japan has seen little change over the past 12 years, and although Taiwan has experienced relatively minor changes, its absolute prevalence remains high.

In 2022, the prevalence rates of weight groups in East Asian countries were as follows. For girls, the prevalence rate of thinness was highest in Taiwan (3.9%), followed by China (3.3%), South Korea (2.2%) and Japan (1.5%). Among boys, Taiwan had the highest thinness rate (5.2%), followed by China (4.0%), Japan (3.0%) and South Korea (2.1%). The prevalence rate of normal weight was highest among girls in Japan (84.8%), followed by China (76.8%), Taiwan (75.5%) and South Korea (73.3%). Similarly, among boys, Japan had the highest normal weight rate (78.0%), followed by China (71.1%), Taiwan (63.7%) and South Korea (55.0%). Overweight and obesity rates were highest regardless of gender in South Korea (girls: 24.6%, boys: 43.0%), followed by Taiwan (girls: 20.5%, boys: 31.0%), China (girls: 19.9%, boys: 24.9%) and Japan (girls: 13.6%, boys: 19.0 (Table 1).

**Table 1:** Prevalence of thinness, normal weight, overweight and obesity in East Asian children and adolescents in 2022.

						(unit: %)
Classification		Standard (WHO)	China	Japan	South Korea	Taiwan
Girls	Thinness (a)	Prevalence of BMI < -2SD	3.3	1.5	2.2	3.9
	Normal(1+2) (b)	Prevalence of -2SD ≤ BMI ≤ 1SD	76.8	84.8	73.3	75.5
	Normal1	Prevalence of -2SD ≤ BMI < -1SD	13.4	12.0	11.8	15.2
	Normal2	Prevalence of -1SD ≤ BMI ≤ 1SD	63.4	72.8	61.5	60.4
	Overweight including obesity(c)	Prevalence of BMI > 1SD	19.9	13.6	24.6	20.5
	Overweight	Prevalence of 1SD < BMI ≤ 2SD	12.2	11.2	16.7	13.8
	Obesity	Prevalence of BMI > 2SD	7.7	2.5	7.9	6.8
	Total (a+b+c)		100.0	100.0	100.0	100.0
Boys	Thinness (a)	Prevalence of BMI < -2SD	4.0	3.0	2.1	5.2
	Normal(1+2) (b)	Prevalence of -2SD ≤ BMI ≤ 1SD	71.1	78.0	55.0	63.7
	Normal1	Prevalence of -2SD ≤ BMI < -1SD	13.2	14.5	9.1	16.9
	Normal2	Prevalence of -1SD ≤ BMI ≤ 1SD	57.9	63.5	45.8	46.9
	Overweight including obesity(c)	Prevalence of BMI > 1SD	24.9	19.0	43.0	31.0
	Overweight	Prevalence of 1SD < BMI ≤ 2SD	9.7	12.7	23.1	16.3
	Obesity	Prevalence of BMI > 2SD	15.2	6.3	19.9	14.7
	Total (a+b+c)		100.0	100.0	100.0	100.0

### Changes in weight distribution by country

We analyzed the changes in the prevalence of weight groups by country over the past 12 years (2010 to 2022) (Table 2). In China, the prevalence rates of thinness (-1.9%p) and normal weight in girls (-5.0%p) have decreased, while the prevalence rates of overweight (2.3%p), and obesity (4.6%p) have increased. For boys, the prevalence rates of thinness (-1.3%p), normal weight (-2.4%p), and overweight (-3.9%p) have decreased, with a marked increase in obesity (7.6%p).

**Table 2:** Differences in the prevalence of thinness, normal weight, overweight including obesity in East Asian children and adolescents between 2010 and 2022.

											(unit: %, %p)	
Classification		Girls					Boys					Dif** (D-C)
		Thinness	Normal			Overweight including obesity (C)	Thinness	Normal			Overweight including obesity (D)	
				Overweight	Obesity				Overweight	Obesity		
China	2022(A)	3.3	76.8	12.2	7.7	19.9	4.0	71.1	9.7	15.2	24.9	5.0
	2010(B)	5.2	81.8	9.9	3.1	13.0	5.3	73.5	13.5	7.7	21.2	8.2
	Dif(A-B)*	-1.9	-5.0	2.3	4.6	6.9	-1.3	-2.4	-3.9	7.6	3.7	N/A
Japan	2022(A)	1.5	84.8	11.2	2.5	13.7	3.0	78.0	12.7	6.3	19.0	5.4
	2010(B)	1.7	85.8	10.1	2.4	12.4	2.8	80.2	12.0	5.1	17.0	4.6
	Dif(A-B)*	-0.2	-1.0	1.1	0.1	1.2	0.2	-2.2	0.7	1.2	2.0	N/A
South Korea	2022(A)	2.2	73.3	16.7	7.9	24.6	2.1	55.0	23.1	19.9	43.0	18.4
	2010(B)	1.7	76.7	16.7	4.8	21.5	1.6	65.0	20.3	13.1	33.4	11.9
	Dif(A-B)*	0.4	-3.4	-0.1	3.1	3.0	0.4	-10.0	2.8	6.8	9.6	N/A
Taiwan	2022(A)	3.9	75.5	13.8	6.8	20.5	5.2	63.7	16.3	14.7	31.1	10.5
	2010(B)	3.1	76.6	14.3	6.0	20.3	3.3	64.3	17.0	15.4	32.4	12.1
	Dif(A-B)*	0.8	-1.1	-0.5	0.8	0.3	1.9	-0.6	-0.7	-0.7	-1.3	N/A

* the difference in prevalence between 2022 and 2010 (%p)

** the difference in the prevalence of Overweight and above between boys and girls (%p)

N/A: Not applicable

Percentage points(%p): percentage points are used to measure the absolute difference between two percentage values.

In Japan, the prevalence rates of thinness (-0.2%p) and normal weight in girls (-1.0%p) have decreased, while the prevalence rates of overweight (1.1%p) and obesity in girls (0.1%p) have increased. For boys, the prevalence of normal weight (-2.2%p) has decreased, and the prevalence rates of thinness (0.2%p), overweight (0.7%p), and obesity (1.2%p) have increased. However, the extent of change is relatively small compared to that of other countries.

In South Korea, the prevalence rates of girls with normal weight (-3.4%p) and overweight (-0.1%p) have decreased, while the prevalence rates of thinness (0.4%p) and obesity (3.1%p) have increased. For boys, the prevalence of normal weight (-10.0%p) has markedly decreased, while the prevalence rates of thinness (0.4%p), overweight (2.8%p), and obesity (6.8%p) have increased. Both boys and girls have seen a noticeable increase in the prevalence rates of overweight and obesity over the past 12 years, resulting in the highest overall rate of abnormal weight.

In Taiwan, the prevalence rates of girls with normal weight (-1.1%p) and overweight (-0.5%p) have decreased, while the prevalence rates of thinness (0.8%p) and obesity (0.8%p) have increased. For boys, the prevalence rates of normal weight (-0.6%p), overweight (-0.7%p), and obesity (-0.7%p) have decreased, while the prevalence of thinness (1.9%p) has increased. Taiwan, like South Korea, has also seen an increase in abnormal weight. (Table 2).

### Trends in gaps between sexes in the prevalence of childhood and adolescent obesity

Across East Asian countries, the prevalence rates of overweight and obesity were relatively higher among boys compared to girls. The gaps in the prevalence of overweight and obesity between girls and boys from 2010 to 2022 were as follows: South Korea (2010: 11.9%p to 2022: 18.4%p), Taiwan (2010: 12.1%p to 2022: 10.5%p), Japan (2010: 4.6%p to 2022: 5.4%p) and China (2010: 8.2%p to 2022: 5.0%p). The gaps have decreased in China and Taiwan but have increased in South Korea and Japan, indicating a widening gap between boys and girls in overweight and obesity prevalence (Table 2).

### The level of increase in the prevalence

We used linear regression analysis to test the level and significance of annual changes in weight groups (Table 3). From 2010 to 2022, there have been significant differences in prevalence among weight groups by most countries and genders, but the differences were not significant for the obesity group of Japanese girls (β = 0.01, p = 0.076) and the overweight group of South Korean girls (β = -0.02, p = 0.118).

**Table 3:** Simple linear regression analysis on the prevalence of weight groups (thinness, normal weight, overweight, obesity) from 2010 to 2022 by country.

		China				Japan				South Korea				Taiwan			
		β	SE(β)	t	p-value	β	SE(β)	t	p-value	β	SE(β)	t	p-value	β	SE(β)	t	p-value
Girls	Thinness	325.69	4.79			38.77	0.43			-70.02	7.03			-128.91	5.46		
	Year(2010–2022)	-0.16	0.00	-67.09	<0.001	-0.02	0.00	-87.19	<0.001	0.04	0.00	10.22	<0.001	0.07	0.00	24.24	<0.001
	Normal	883.98	11.63			258.63	29.89			633.66	7.50			246.60	10.13		
	Year(2010–2022)	-0.40	0.01	-69.21	<0.001	-0.09	0.01	-5.79	<0.001	-0.28	0.00	-74.50	<0.001	-0.08	0.01	-16.82	<0.001
	Overweight	-347.76	34.04			-179.70	19.59			53.98	21.88			105.77	1.35		
	Year(2010–2022)	0.18	0.02	10.55	<0.001	0.09	0.01	9.71	<0.001	-0.02	0.01	-1.69	= 0.118	-0.05	0.00	-68.21	<0.001
	Obesity	-761.91	20.77			-17.70	10.23			-517.62	17.86			-123.46	4.83		
	Year(2010–2022)	0.38	0.01	36.93	<0.001	0.01	0.01	1.95	= 0.076	0.26	0.01	29.32	<0.001	0.06	0.00	26.89	<0.001
	Overweight including obesity	-1109.67	14.84			-197.40	29.82			-463.64	6.96			-17.69	4.84		
	Year(2010–2022)	0.56	0.01	75.88	<0.001	0.10	0.01	7.05	<0.001	0.24	0.00	69.97	<0.001	0.02	0.00	7.87	<0.001
Boys	Thinness	221.00	10.33			-43.06	1.78			-77.32	5.19			-325.88	16.68		
	Year(2010–2022)	-0.11	0.01	-20.96	<0.001	0.02	0.00	25.78	<0.001	0.04	0.00	15.23	<0.001	0.16	0.01	19.78	<0.001
	Normal	362.42	77.22			466.99	60.95			1727.07	82.02			148.38	27.09		
	Year(2010–2022)	-0.14	0.04	-3.77	= 0.003	-0.19	0.03	-6.35	<0.001	-0.83	0.04	-20.32	<0.001	-0.04	0.01	-3.10	<0.001
	Overweight	745.28	86.38			-124.76	21.98			-436.60	26.81			129.20	3.06		
	Year(2010–2022)	-0.36	0.04	-8.48	<0.001	0.07	0.01	6.23	<0.001	0.23	0.01	17.10	<0.001	-0.06	0.00	-36.80	<0.001
	Obesity	-1228.70	10.59			-199.16	39.90			-1113.16	103.71			-123.46	4.83		
	Year(2010–2022)	0.62	0.01	117.09	<0.001	0.10	0.02	5.12	<0.001	0.56	0.05	10.88	<0.001	0.06	0.00	26.89	<0.001
	Overweight including obesity	-483.42	87.43			-323.92	61.63			-1549.76	77.24			277.49	12.78		
	Year(2010–2022)	0.25	0.04	5.80	<0.001	0.17	0.03	5.54	<0.001	0.79	0.04	20.55	<0.001	-0.12	0.01	-19.24	<0.001

β, beta coefficient used to measure and predict the relationship between variables (dependent variable: prevalence; independent variable: year).

SE, standard error used to evaluate the precision of an estimate.

t, t-value used to assess the statistical significance of each regression beta coefficient (β)

A p-value less than 0.05 indicate statistical significance, typically aligning with a 95% confidence interval.

For Chinese girls, the prevalence rates by weight group were thinness (β = -0.16, p<0.001), normal weight (β = -0.40, p<0.001), overweight (β = 0.18, p<0.001), and obesity (β = 0.38, p<0.001). For Chinese boys, the rates were thinness (β = -0.11, p<0.001), normal weight (β = -0.14, p<0.001), overweight (β = -0.36, p<0.001), and obesity (β = 0.62, p<0.001). Both girls and boys in China have shown a sharp increase in obesity prevalence.

Among Japanese girls, the prevalence rates of thinness (β = -0.02, p<0.001) and normal weight (β = -0.09, p<0.001) have decreased, while the prevalence rate of overweight (β = 0.09, p<0.001) has increased. Among boys, the prevalence rates of thinness (β = 0.02, p<0.001), overweight (β = 0.07, p<0.001), and obesity (β = 0.10, p<0.001) have increased, while the prevalence rate of normal weight (β = -0.19, p<0.001) has decreased. Although there have been no substantial changes overall, a decrease in normal weight and an increase in obesity were observed among boys.

South Korean girls have shown increases in thinness (β = 0.04, p<0.001) and obesity (β = 0.26, p<0.001), and a decrease in normal weight (β = -0.28, p<0.001). South Korean boys have also exhibited increases in thinness (β = 0.04, p<0.001), overweight (β = 0.23, p<0.001), and obesity (β = 0.56, p<0.001), and a sharp decrease in normal weight (β = -0.83, p<0.001).

Among girls in Taiwan, the prevalence rates of thinness (β = 0.07, p<0.001), normal weight (β = -0.08, p<0.001), overweight (β = -0.05, p<0.001), and obesity (β = 0.06, p<0.001) were confirmed. Among boys, the prevalence rates of thinness (β = 0.16, p<0.001), normal weight (β = -0.04, p<0.001), overweight (β = -0.06, p<0.001), and obesity (β = 0.06, p<0.001) were found. Overall, there have been no notable changes, but an increase in thinness among boys and a decrease in other weight groups were noted.

The prevalence rate of thinness among girls in East Asia has increased in Taiwan and Korea but has decreased in China and Japan. Among boys, the prevalence rate of thinness has increased in Taiwan, Korea, and Japan, but has decreased in China. The changes in the prevalence rate of thinness were more significant in Taiwan and China. The prevalence rates of overweight and obesity among girls have increased each year in the order of China, Korea, Japan, and Taiwan. Among boys, the prevalence rate has increased in the order of Korea, China, and Japan, excluding Taiwan (β = -0.12, p<0.001).

### Changes in the contribution of age to the prevalence

**Fig 2:**
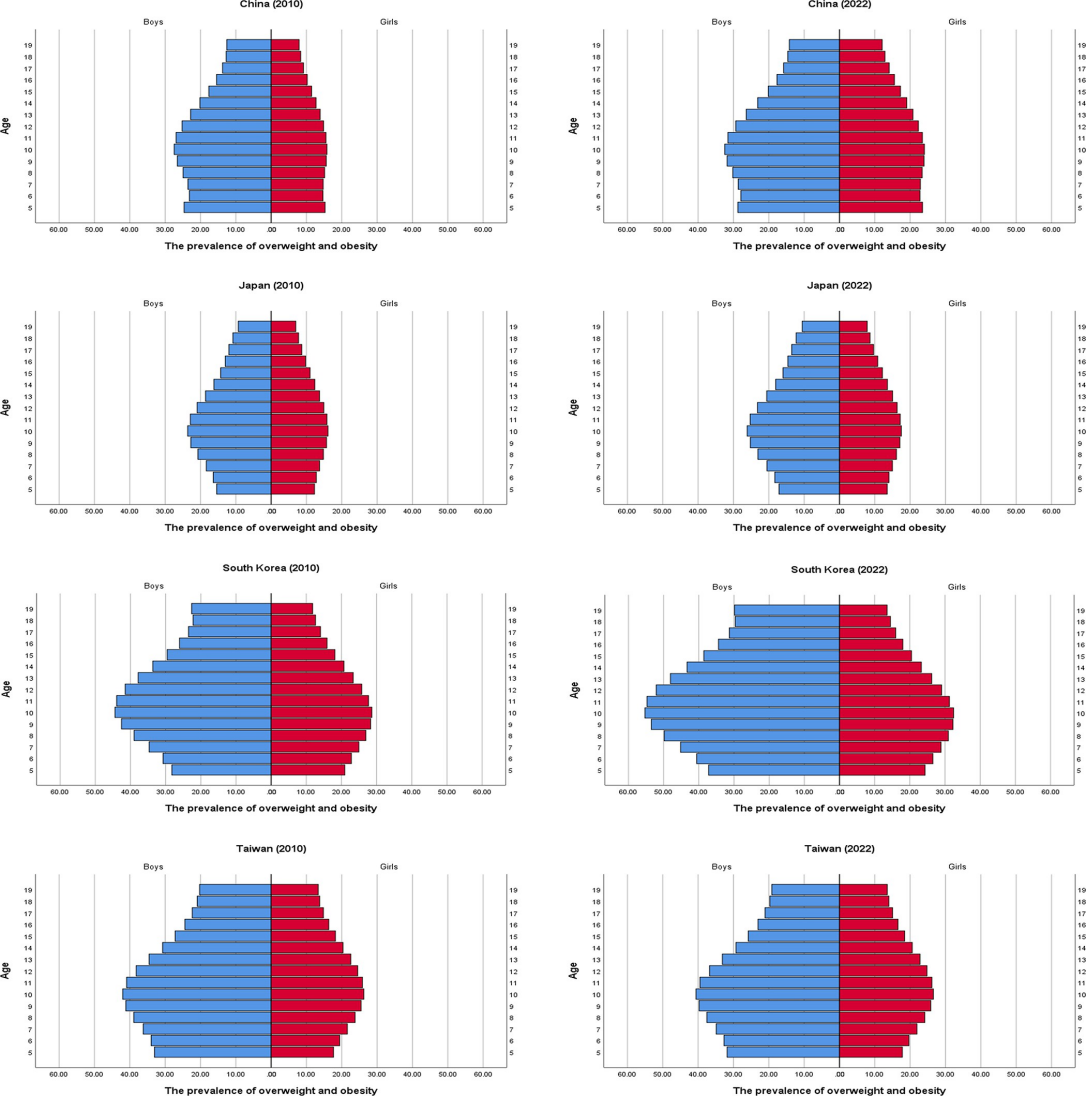
The pyramid of overweight and obesity prevalence by age (2010 vs. 2022).

In China, the prevalence rates of overweight and obesity were relatively evenly distributed across age groups in 2010, but by 2022, the prevalence rates had increased among 10 to 15-year-olds and decreased among 17 to 19-year-olds. In Japan, although the prevalence rates of overweight and obesity have increased, there were no notable differences in prevalence distribution across age groups. In South Korea, the prevalence rates of overweight and obesity have risen across most age groups, with a greater increase in boys than in girls. Notably, the prevalence rate in girls aged 15 to 19 has manifested a substantial rise. In Taiwan, although the absolute prevalence rates of overweight and obesity were not low, there were no remarkable differences in age-specific prevalence between 2010 and 2022. Interestingly, the prevalence rates of overweight and obesity were the highest in children and adolescents aged 10 to 11 years for both girls and boys across all countries. (Fig 2).

## Discussion

According to the WHO fact sheets, the prevalence of overweight (including obesity) among children and adolescents aged 5 to 19 had risen dramatically from 8% in 1990 to 20% in 2022 worldwide. The rise has occurred similarly among both boys and girls [7]. Pediatric obesity does not naturally resolve as the child grows. However, it instead persists into adulthood, with various physical and psychological complications before adulthood, and strongly predicts adult weight status and future health outcomes [8].

Underweight as well as obesity can lead to adverse health outcomes, both immediate and long-term. This condition is associated with poor academic performance, impaired physical growth and developmental milestones, anemia, respiratory failure, wound complications, extended hospital stays, and increased vulnerability to infections and diseases [9]. Persistent thinness during adolescence is associated with an increased risk of adverse health outcomes, such as chronic diseases, impaired physical development, and an increased risk of all-cause mortality later in life [10]. The double burden of malnutrition (DBM), defined as the coexistence of both undernutrition and overnutrition in the same population within communities, families, and even individuals across the life course, continues to be a major global public health problem [11, 12]. The DBM occurs at an individual level, such as in cases in which children with undernutrition are later exposed to high-calorie food and develop obesity [13]. The growing evidence of the DBM suggests that comprehensive policy efforts are needed to address the issues of undernutrition and obesity simultaneously [14]. In almost all countries for adults, school-aged children, and adolescents, the increases in double burden were driven by increases in obesity and decreases in double burden by declining underweight [5]. Analyzing the trends in childhood obesity and underweight is crucial for developing effective preventive and management strategies for children’s health.

The people in the regions of East Asia, including Chinese, Japanese, South Korean, and Taiwanese, belong to the Mongolian race [15]. Chinese, Japanese, South Korean, and Taiwanese share certain physical and genetic characteristics because of their geographical proximity and historical interactions and exhibit similar physical characteristics and disease susceptibilities.

The pronounced differences in the Asian population include the high proportion of body fat and predisposition to visceral fat accumulation and subsequent abdominal obesity compared with Caucasians, leading to an increased risk of diabetes at lower BMI levels in Asians [16, 17]. However, each country has different genetic backgrounds and distinct cultural variations, including dietary patterns and physical activity levels influenced by lifestyle, education, and urbanization, while healthcare infrastructure, preventive care, and government initiatives also significantly affect obesity prevalence. In Japan, since the late 2010s, annual percentage changes of pediatric obesity have significantly increased in 12 to 14-year-old boys (6.7%–8.9%) and girls of many age groups (2.6%–8.6%) [18]. In South Korea, the prevalence rate of obesity in children and adolescents surged from 9.7% in 2012 to 19.3% in 2021, with a greater increase in boys [19]. China, a vast country with regional disparities in socio-economic conditions, dietary habits, and lifestyles, has remarkable regional differences in obesity rates among children and adolescents [20]. The prevalence of overweight and obesity among children and adolescents aged 7 to 18 years had soared from 5.3% in 1995 to 24.2% in 2019, affecting almost a quarter of children and adolescents in China [21]. In Taiwan, the prevalence of obesity had increased from 13.19% in 2009 to 14.97%, and the prevalence of overweight had declined from 13.17% in 2009 to 12.63% in 2018 [22]. The use of country-specific growth charts in diagnosing childhood obesity is essential due to variations in growth patterns among children and adolescents in different countries. China, Japan, South Korea, and Taiwan have developed their growth charts tailored to their populations [23–26]. Thus, this study aimed to analyze the changes in weight distribution and compare the prevalence and trends of pediatric obesity in East Asian countries using WHO criteria.

Our analysis revealed significant shifts in the prevalence of underweight, normal weight, overweight, and obesity in East Asian children and adolescents from 2010 to 2022. Overweight and obesity have been on the rise in East Asia, with particularly rapid increases in South Korean boys and Chinese girls. In 2022, the overweight and obesity rates were the highest in South Korea (girls: 24.6%, boys: 43.0%). The sharp rise in obesity among South Korean boys and Chinese girls is alarming and underscores the need for targeted public health policies and reassessment of current management strategies. Japan exhibited the least change in overweight and obesity rates. Taiwan showed a less pronounced change, although the absolute prevalence remains high, indicating a potential stabilization but not necessarily an improvement in public health outcomes. The traditional Japanese diet, characterized by its abundance of seafood, vegetables, and fermented soy products, coupled with its low consumption of calorie-dense, minimally seasoned foods, frequent home-cooked meals, and small portion sizes, along with the distinctive divided meal system, may contribute to the notably low obesity prevalence in Japan [27]. Moreover, according to a comparative analysis of physical activity in East Asian countries from 2016 to 2022, South Korea and Taiwan had experienced no changes in overall physical activity, while China had shown improving levels of physical activity, with no notable changes in governmental policies. Japan exhibited improvements in physical fitness, active transportation, community, and the environment, along with stable levels of sedentary behavior, with moderate governmental policies throughout the period [28].

The prevalence rates of overweight and obesity have consistently been higher in boys than in girls across all countries. The gap between the sexes widened significantly in South Korea and Japan. For instance, the gap in South Korea had increased from 11.9%p in 2010 to 18.4%p in 2022. In high and upper-middle-income countries, boys tend to have higher obesity prevalence than girls, but the possible reasons and implications of this difference are yet to be fully understood [29]. The difference in prevalence of childhood and adolescent obesity between sexes may be driven by biological and sociocultural influences, like body composition and gender-based stereotypes [29, 30]. Thinness has also been an issue in East Asia, with notable increases observed in Taiwan and South Korea. The increase in thinness in Taiwan is particularly significant, suggesting a growing public health concern. In contrast, China had exhibited a decrease in thinness among both girls and boys. While China shows a pattern of increased obesity accompanied by decreases in underweight, normal weight, and overweight categories, South Korea exhibits a pattern in which the increase in obesity is accompanied by an increase in underweight prevalence. In Taiwan, the increase in obesity had not been as rapid as exhibited in other countries; however, similar to South Korea, there had been an accompanied increase in the prevalence of underweight individuals. Such discrepancy among countries highlights the need for country-specific strategies. South Korea and Taiwan need to focus on preventive and management policies for DBM. China, on the other hand, may need to concentrate more on policies addressing the increasing rates of overweight and obesity.

The prevalence rates of children and adolescents with normal weight have decreased across all countries. Out of all weight categories, the normal weight rate was the highest, suggesting relatively effective weight management strategies among East Asian countries. Conversely, Korea had displayed a sharp decline in normal weight prevalence in boys (-10.0%p), indicating an increasing trend towards either underweight or overweight and obesity. This shift is a growing public health issue that requires urgent interventions to promote healthy weight management. Healthy weight initiatives should approach the weight spectrum as a whole, not focusing on certain weight categories [10].

Comparing the distribution of obesity among age groups can pinpoint the ages most vulnerable to obesity, allowing tailored interventions for susceptible age groups. Though there has been a lack of reports on childhood obesity that classify age groups in a one-year interval, some studies have utilized a relatively short interval span of five years. In a US study, data were classified by sex and age (2 to 5, 6 to 11, and 12 to 19 years). From 2003 to 2004 and from 2007 to 2008, the 6-to-11 age group had the highest obesity prevalence, whereas from 2005 to 2006, the 12-to-19 age group had the highest obesity prevalence [31]. In a study conducted in China, the highest prevalence of obesity was observed in 12-year-olds for both sexes, suggesting a need for interventions before 12 years of age [32]. According to another study conducted in West Asia, the prevalence of obesity was the highest in the youngest age group (2 to 6 years), followed by the oldest age group (14 to 19 years); the lowest was in children aged 7 to 13 years [33]. Our study showed the prevalence of overweight and obesity has been the highest in children aged 10 to 11 years in all countries. It is important to note that the diagnostic criteria for overweight and obesity in adults in Asia (BMI 23 and above) do not align with the criteria for overweight and obesity in children and adolescents (BMI at or above the 85th percentile). This discrepancy leads to an underestimation of obesity rates in late adolescence, further emphasizing the necessity of treatment of childhood overweight and obesity. Our results, thus, suggest that the normalization of body weight before the onset of puberty is crucial.

There are some limitations in our study. First, estimating the prevalence rate of pediatric obesity using a national reference may better capture the prevalence rates of overweight and obesity in each country. Though our study calculated obesity prevalence based on WHO standards for comparison among nations, there may be a difference from the obesity prevalence calculated using the growth standards of each country. Second, to compare the prevalence rate of obesity across multiple countries over time, studies must consider various factors at multiple levels, from a national level, such as national policies and childhood obesity programs curriculum, to an individual level, such as socioeconomic factors, parental education, and nutritional supplies. However, due to limitations in the available data, analyzing such demographic factors was challenging.

Despite the limitations of this study, to the best of our knowledge, our study is the first to compare the recent prevalence of pediatric obesity and the change in weight distribution in East Asian countries, utilizing uniform criteria.

The trends in the prevalence of thinness, normal weight, overweight, and obesity among East Asian children and adolescents underscore a growing public health challenge. The significant increases in overweight and obesity, particularly among South Korean boys and Chinese girls, are alarming and call for urgent public health interventions. Attention should also be paid to the trend of increasing thinness of children and adolescents in South Korea and Taiwan. Additionally, the declining proportion of children with a healthy weight in all countries is another concerning issue. Addressing these trends will require a multifaceted approach, including promoting healthy dietary habits, increasing physical activity, and implementing effective public health policies. The varying trends across different countries and gender groups also highlight the importance of tailored strategies to address the unique challenges faced by each population segment. Considering the age distribution of obesity, intervention for obesity would be preferable before the age of 10 to 11. Further research is essential to explore the underlying causes of these trends and develop targeted interventions that can effectively combat childhood and adolescent weight issues in East Asia.
